# Design and Performance of an Email-Based Patient Recruitment Campaign in Primary Care Research: Formative Secondary Analysis

**DOI:** 10.2196/67088

**Published:** 2026-03-06

**Authors:** Vanessa T Vaillancourt, Marie-Dominique Poirier, Amélie Fournier, Sabrina T Wong, Marie-Eve Poitras

**Affiliations:** 1 Department of Family Medicine and Emergency Medicine, Université de Sherbrooke, 3001 12 Ave N Immeuble X1, Sherbrooke, QC, J1H 5N4, Canada, 1 4185415050 ext 203239; 2 Applied Science and Medicine, School of Nursing, University of British Columbia, Vancouver, BC, Canada; 3 Centre for Health Services and Policy Research, School of Nursing, University of British Columbia, Vancouver, BC, Canada

**Keywords:** web-based survey, primary care, email-based recruitment, patient recruitment, engagement metrics

## Abstract

**Background:**

Recruiting patients in primary care research remains challenging due to clinical workload, staffing constraints, and the need to limit disruption to routine care. Traditional recruitment methods often place a substantial burden on clinics, prompting research teams to adopt low-burden and scalable approaches such as email-based recruitment. Despite its growing use, limited empirical evidence describes how email recruitment campaigns are designed and how they perform when targeting primary care patients in real-world settings.

**Objective:**

This study aims to descriptively examine engagement metrics from an email recruitment campaign targeting primary care patients.

**Methods:**

We conducted a formative, descriptive secondary analysis of engagement metrics generated during a large-scale email recruitment campaign conducted as part of the Quebec component of the Patient-Reported Indicator Survey-Organisation for Economic Co-operation and Development survey. Between June 2023 and January 2024, a total of 12 primary care clinics invited eligible adult patients (aged ≥45 years) to complete an online survey using a standardized email template distributed via an email marketing platform. Collected engagement metrics included delivery rates, open rates, click-through rates, conversion rates, and device type. Analyses were descriptive and conducted at the clinic level.

**Results:**

For 15,277 patients, invitations were successfully delivered to 14,758 (96.6%). The mean open rate for the initial invitation was 73.4% (10,828/14,757; range 57%‐88%), decreasing with reminders. Most emails were opened on computers (25,868/30,279, 85.4%). Out of a total of 445 emails, 42 (9.4%) were undelivered due to technical issues, and 403 (90.6%) were undelivered due to incorrect email addresses. The overall conversion rate was 9.7% (1430/14,758). Click-through rates varied by content, with the highest engagement observed for the survey link and lower engagement for supplementary video materials. Reminder emails substantially increased survey participation across clinics (759/1138, 66.7%). Participants who completed the questionnaire were predominantly aged 60 to 69 years, identified as female, and had completed postsecondary education.

**Conclusions:**

This formative analysis suggests that email-based recruitment is a feasible and low-burden approach for engaging primary care patients in research. Engagement metrics offer valuable insights at the implementation level to inform the design, adaptation, and monitoring of digital recruitment strategies in real-world primary care settings. These findings provide practical, implementation-oriented insights to inform the design, refinement, and evaluation of email recruitment campaigns in primary care research.

## Introduction

The increasing administrative burden and the insufficient workforce place significant pressure on Canadian primary care clinics [1,2]. The schedules of family physicians in primary care are overloaded, and their participation in research initiatives can be hindered by numerous factors, including mismatches between their needs and the research objectives, staff shortages, administrative burdens, disruptions to clinical routines, and additional documentation requirements [3]. Consequently, their participation in research initiatives must be made as easy and straightforward as possible. Recruiting patients for research participation in clinics is highly challenging [4]. If clinic staff are required to dedicate time to explaining the initiative and responding to patients’ questions, this fragments their clinical practice and disrupts the routine workflow. While this added burden is unavoidable, it necessitates the use of low-burden recruitment methods, moving away from traditional on-site or mail-based approaches. Despite many clinics showing interest in participating in research, engaging them remains challenging due to complex procedures, extensive paperwork, staffing shortages, and insufficient office space for recruitment and consent [3,5,6]. Ethical issues concerning the initial contact with patients are also at stake because it “should be made by someone that individuals would expect to have relevant information about them, or in other ways that do not inappropriately intrude on their life or privacy*”* [7].

Nearly 29 million Canadians use email, with approximately 20% opening marketing emails [8]. As a result, email has become a convenient and efficient method for recruiting research participants across various fields [4,9]. Recruitment emails typically outline the research purpose, potential benefits for participants, and any compensation or incentives that will be offered. Combined with a web-based survey, email recruitment enables primary care researchers to quickly reach a broad audience and track responses, while being less resource intensive than in-clinic or phone recruitment.

Beyond initial recruitment, sustaining participant engagement amidst the myriad of digital stimuli remains challenging. Capturing and maintaining the interest and commitment of potential participants requires deploying specific strategies. Innovative approaches grounded in empirical research are crucial for achieving optimal participant recruitment and engagement outcomes in navigating this complex landscape. Numerous factors have been shown to influence response rates, including shorter survey length, providing incentives, high relevance of the content and survey topic, and attractive visual display [10]. However, there is a lack of information on how patients in primary care engage when invited to participate in research via email. Most existing studies focus on email recruitment of health care professionals [11-13], and those describing email content in sufficient detail or using marketing platforms to monitor engagement metrics are rare.

In recent years, research teams have increasingly relied on email as a recruitment strategy, underscoring the need to better document how such campaigns are designed and how they perform in real-world settings. Accordingly, this study aims to describe the implementation and performance of an email-based recruitment campaign targeting primary care patients using engagement metrics collected from a large-scale survey. The findings are intended to provide practical, implementation-oriented insights to inform the design, adaptation, and monitoring of future email recruitment strategies in primary care.

## Methods

### Overview

This secondary analysis followed a prespecified descriptive analytic plan focused on characterizing engagement patterns at the clinic level. Primary outcomes included email delivery, open rates, click-through rates, device type, and conversion rates. No hypothesis testing was conducted, as the study objective was exploratory and formative. This paper presents data from reports and indicators gathered during the Patient-Reported Indicator Survey (PaRIS) national survey in Quebec, Canada. The PaRIS is an international survey led by the Organisation for Economic Co-operation and Development (OECD) to develop, standardize, and implement a new generation of indicators that measure the outcomes (patient-reported outcome measures [PROMs]) and experience of health care (patient-reported experience measure [PREMs]) most relevant to primary care patients worldwide. The PaRIS initiative has been described in detail elsewhere [14,15]. The OECD provided guidance on patient recruitment across participating countries, enabling each country to tailor its recruitment methods to its chosen approach, whether by phone, mail, email, or in-person at clinics. Quebec opted to recruit patients through a large-scale email campaign. The sample consisted of patients attending primary care clinics participating in the Canadian PaRIS-OECD national survey in the province of Quebec. The inclusion criteria for patients were as follows: (1) being 45 years or older, (2) having consulted their primary care clinician within the last 6 months, and (3) providing an email address. In accordance with the OECD’s guidelines for the PaRIS study, the targeted sample size was established at 75 patients per clinic, although clinics could recruit more if feasible [15].

Our team, comprising a registered nurse and researcher, a research coordinator, a patient partner, and a scientific graphic designer, cocreated an invitation template for patient recruitment. STW and MEP are coleads for PaRIS-OECD in Canada. First, we identified the essential items to include in the email invitation. We then drafted the initial content, refining it before the scientific graphic designer further refined it to enhance its visual clarity and usability. As Canada has 2 official languages, French and English, we created bilingual email recruitment materials.

Building on the Canadian Institutes for Health Research’s strategy for patient-oriented research [16] and our previous findings, which demonstrated that recruitment facilitated by a patient partner results in a higher participation rate, we incorporated a patient partner video clip in the email invitation. This 1-minute 50-second French video, featuring English subtitles, showcased a patient partner explaining the project’s objectives and emphasizing the importance of participation. The scientific graphic was produced using Adobe Premiere Pro (version 25.4). The email template (Multimedia Appendix 1) was designed using the online marketing platform CyberImpact 2025, which provides delivery and engagement metrics and contains the following 5 sections:

1. Title of the study and catchy wording
2. Greetings and invitation purpose, including an introduction to the patient partner video and a link to it (research team’s YouTube channel)
3. Links to French and English PaRIS study questionnaires and contact information to obtain support from the research team
4. Aim of the study, participants’ contribution, and what is in it for participants
5. Two links leading to a video about PREMs and PROMs (research team’s YouTube channel), previously produced by the research team, and greetings

We also created a reminder template with adjusted wording to include the reminder element and remove links to video clips discussing PREMs and PROMs, thereby streamlining the invitation template. Finally, we created support materials for participating clinics, including a PDF illustrating the steps for importing emails into the marketing platform and how to schedule email campaigns and several examples of email subjects such as “PaRIS Study-Survey on your care experience at [name of the clinic],” “[name of the clinic]: PaRIS research project,” and “We need your opinion, please help us!” Each clinic was instructed to program 1 initial invitation and 3 reminders, all scheduled to be sent at 6 AM.

Participating primary care clinics obtained data from the statistics provided by CyberImpact and shared anonymized reports with the research team, as approved by the ethics committee. The following categories of data were used: mailing performance, opening statistics, and statistics on embedded links. Briefly, email open and click rates were measured using hidden tracking pixels. Specifically, the pixel was activated only when the images embedded in the email were downloaded, which helped reduce false openings generated by automated systems that scanned messages. A certain margin of error remained, for example, due to image blocking or user privacy settings. However, CyberImpact considers the reliability of these measures to be very high, estimating data accuracy at approximately 95%. We used the analytic data from YouTube to track metrics (number of views, average view duration, and average percentage of video viewed) for the patient partner introduction video.

The open rate was calculated as the percentage of patients who opened the email out of the total number of patients who received the email. The click-through rate refers to the percentage of people who click a link in an email out of those who open it. The conversion rate is the number of recipients who take the desired action compared with the number of recipients who received the email. The unsubscribe rate refers to the number of patients who unsubscribe out of the number of patients who received the email [17]. Quantitative data were analyzed using descriptive statistics, including measures of central tendency (mean) and variability (SD) using a 95% CI, as well as the minimum and maximum values of the variables’ means across clinics, using SPSS (IBM Corp) software. We did not use a reporting guideline. Instead, we emphasized transparency in describing the data sources, metrics examined, unit of analysis, and descriptive analytic approach, consistent with the exploratory objectives of the study and the reporting standards commonly used for formative and implementation-focused research.

### Ethical Considerations

This study was in accordance with the ethical standards of the responsible committee on human experimentation and with the World Medical Association Declaration of Helsinki. Ethics approval was obtained from the research ethics board of the Ministry of Health and Social Services of the province of Quebec (CCER-22-23). Patients gave their consent to participate in the PaRIS-OECD national study, and this consent covered secondary analysis. Patients participating in the PaRIS study in Quebec were eligible for a draw to win one of three US $25 grocery gift cards among respondents from their clinic. Demographic characteristics are presented in aggregate, and no identifiable participant information is included in this study.

## Results

Between June 2023 and January 2024, a total of 12 primary care clinics participated in the PaRIS-OECD national survey in the province of Quebec. The research coordinator met with a staff member from each participating clinic to assist in programming and sending email invitations and reminders to patients. The clinic staff member then chose their preferred formulation for the email subject, either by creating their own subject line or using 1 of the provided examples. The clinic staff sent a first invitation to patients the day after the meeting. Email distribution was scheduled to accommodate each clinic’s operational context, including avoidance of public holidays and alignment with concurrent recruitment activities. All clinics expressed that 3 reminders were excessive and that they did not want to oversolicit their patients. Accordingly, only 2 reminders were sent to comply with this request. All invitations and reminders were sent on weekdays at 6 AM, except for 1 clinic that preferred to send them at 8 AM to coincide with its opening hours.

Twelve primary care clinics sent the initial email invitation to 15,277 patients. Across clinics, 14,758 (96.6%) patients received the email invitation in their inbox, while 14,662 (96%) patients received the first reminder email, and 14,640 (95.8%) patients received the second reminder email. The number of invited patients per practice varied from 47 to 4366 (median 416.5, IQR 143.5-945). Combining the initial invitation and the 2 reminders, a total of 44,771 emails were sent, and 98.4% (n=44,060) were delivered. Patients who had already initiated or completed the questionnaire still received the reminder emails. A total of 629 emails were undelivered due to technical issues (n=119, 18.9%) or an incorrect email address (n=510, 81.1%). A total of 176 patients unsubscribed from the email invitation, corresponding to an unsubscribe rate of 1.2% (176/14,758). Patients provided some reasons explaining their unsubscription; 48 (27.3%) patients unsubscribed because they never subscribed to the emailing list, 34 (19.3%) patients unsubscribed because they no longer wished to receive the communications, and 13 (7.4%) patients unsubscribed because they received too many emails. A total of 7 (4%) patients reported that they did not like the content. Of the 176 patients, 76 (43.2%) patients who unsubscribed did not provide a reason. A total of 0.3% (39/15,277) of patients contacted the research team by phone or email to obtain more information, validate their eligibility, or seek support with the web-based survey. Among them, 12 (30.7%) asked for a paper copy of the questionnaire.

For the initial invitation, the open rate was 73.4% (10,828/14,758), with a range of 57% to 88% (SD 6.57) across clinics. For the first reminder, the open rate dropped to 68% (9968/14,662; range 52%-86 %; SD 8.51), and for the last reminder, the open rate was 64.8% (9483/14,640), with a range of 47% to 84% (SD 10.09). Of the 30,279 opened emails, most invitation and reminder emails (n=25,868, 85.4%) were opened on a computer, 3803 (12.6%) invitation and reminder emails were opened on a mobile phone, and 643 (2.1%) invitation and reminder emails were opened on a tablet. The information was missing for 19 (0.1%) emails.

The highest click-through rate was 16% (range 7%‐27%), observed with the first reminder and linked to the French questionnaire. The video clip featuring the patient partner obtained the highest click-through rate in the first email invitation (8%; range 1-19) and was viewed 1700 times (direct URL source). The average view duration was 1 minute and 20 seconds, and the average percentage of the video viewed was 73%. The initial invitation and reminders were viewed 863 times in a web browser. Table 1 lists the click-through rates for each link included in the invitations.

**Table 1.** Click-through rate for invitation sections.

Link to	Click rate^a^ (range)^b^ for the first email (n=10,828)	Click rate (range) for the first reminder (n=9968)	Click rate (range) for the second reminder (n=9483)
Patient partner video clip	8.1% (1.2%-18.9%)	5.3% (0.8%-11.7%)	4.0% (0.0%-7.8%)
French Patient-Reported Indicator Survey questionnaires	15.2% (5.3%-25.7%)	16.6% (6.8%-27.5%)	13.9% (3.8%-20.0%)
English Patient-Reported Indicator Survey questionnaires	2.1% (0.0%-14.9%)	0.6% (0.0%-2.9%)	0.4% (0.0%-2.2%)
View invitation in a web browser	4.8% (0.8%-7.1%)	2.8% (0.0%-9.5%)	4.0% (0-8.6%)
Video clip about patient-reported experience measures	1.3% (0.0%-2.7%)	N/A^c^	N/A
Video clip about patient-reported outcomes measures	0.6% (0.0%-2.7%)	N/A	N/A

a Number of patients who clicked on the link/number of patients who opened the invitation.

b Minimum and maximum click rate across clinics.

c N/A: not applicable. The link to the video clip was not included in the reminders.

In this study, the conversion rate was 9.7% (1430/14,758), defined as the proportion of patients who initiated the questionnaire (n=1430) among those who received the initial email invitation (n=14,758).

**Figure 1.**
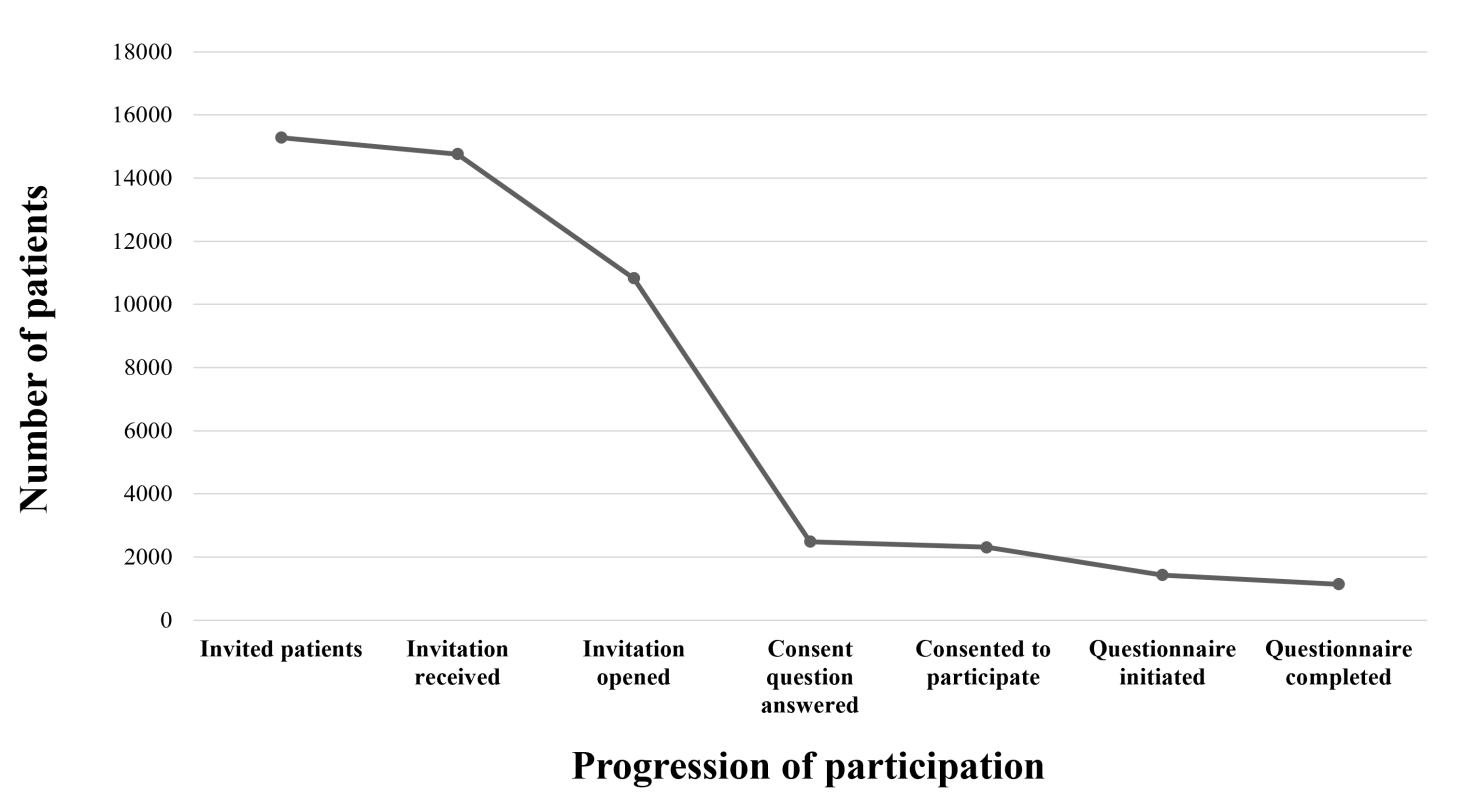
Recruitment and retention across study phases.

Figure 1 illustrates the flow of recruited patients from the initial email invitation to the completion of the web-based survey. Across the initial invitation and the 2 reminders, the emails were opened 30,279 times; however, it was not possible to determine whether these were unique individuals, as the same person could have opened both the initial invitation and 1 or more reminder emails. A total of 2486 patients opened the Research Electronic Data Capture (REDCap; Vanderbilt University) survey and answered the consent question. Among them, 2307 (92.8%) patients provided their consent to participate in the study, and 1430 (62%) initiated the questionnaire. From them, 1138 (79.6%) patients completed all the included items.

Across clinics, the first reminder was sent on average on day 10 (range across clinics: 7‐16 days from initial invitation), and the second reminder was sent on average on day 22 (range across clinics: 3-22 days after the first reminder). Across clinics, questionnaire completion rates increased with the use of reminder emails, with 379 (2.6%) out of 14,758 questionnaires completed after the initial invitation, 437 (3%) out of 14,662 after the first reminder, and 322 (2.2%) out of 14,640 after the second reminder (Table 2). Completion rates varied substantially between clinics, and the inclusion of reminder emails resulted in an approximate 200% (759/379) increase in the cumulative number of completed questionnaires.

**Table 2.** Questionnaire completion per clinic after initial invitation and reminders.

Clinic number	Patients who completed the questionnaire after receiving the initial invitation, % (n/N)	Patients who completed the questionnaire after receiving the first reminder, % (n/N)	Patients who completed the questionnaire after receiving the second reminder, % (n/N)
2401	1.7 (16/966)	2.3 (22/955)	4 (34/949)
2402	3.4 (47/1364)	4.6 (63/1361)	2.5 (34/1356)
2403	3.9 (7/178)	2.2 (4/178)	1.1 (2/177)
2405	5 (42/849)	4.8 (41/850)	3.1 (26/850)
2407	3.6 (30/838)	5.0 (42/833)	4.3 (36/832)
2408	5.2 (25/481)	4.6 (22/476)	4.8 (23/476)
2409	3.8 (87/2272)	4.5 (103/2271)	3.4 (78/2264)
2410	3.6 (46/1273)	4 (50/1267)	2.4 (31/1265)
2414	0 (0/83)	7.2 (6/83)	7.2 (6/83)
2418	2.4 (45/1908)	2.8 (53/1903)	1.4 (27/1900)
2419	0.7 (29/4366)	0.6 (24/4348)	0.4 (19/4310)
2420	2.8 (5/179)	5.1 (7/137)	3.4 (6/178)
Total	2.6 (379/14,758)	3 (437/14,662)	2.2 (322/14,640)

We examined the characteristics of patients who completed the sociodemographic section of the questionnaire (Table 3). Respondents were mainly aged between 60 and 69 years, identified as female, had a college or university diploma, and were of White ethnicity.

**Table 3.** Sociodemographic characteristics of primary care patients who completed the sociodemographic section of the questionnaire (N=1426).

Characteristics	Patients, n (%)
Age (years)	
<45^a^	118 (8.3)
45‐49	139 (9.7)
50‐69	287 (20.1)
60‐69	485 (34.0)
≥70	397 (27.9)
Gender	
Men	444 (37.9)
Women	723 (61.8)
Nonbinary	3 (0.3)
Education	
Partial high school or less	60 (4.2)
High school diploma	191 (13.4)
Partial college	123 (8.6)
College or university degree	1027 (72.1)
Missing	25 (0.02)
Ethnicity^b^	
Black	16 (1.4)
Métis, Inuit, and First Nations	15 (1.3)
White	1098 (93.8)
Other	27 (3.5)

a One clinic made a mistake when extracting patients’ email addresses. Patients aged younger than 45 years received the invitation, and some patients responded to the questionnaire, despite being ineligible for the study.

b Ethnicity categories were determined by the Organisation for Economic Co-operation and Development and reported as found in the questionnaire.

## Discussion

### Principal Findings

Overall, this formative secondary analysis provides practical insights into how primary care patients engage with email-based research invitations in real-world clinical contexts. Our findings suggest that email invitations are an efficient and effective method for reaching a large number of primary care patients. High delivery and open rates suggest that emails sent on behalf of trusted primary care clinics are effective at reaching patients. Moreover, conversion rates indicate that a meaningful proportion of recipients proceeded to survey participation. These findings support the feasibility of email recruitment as a low-burden approach for clinics and research teams. These results lead us to the following observations: (1) email is a highly feasible recruitment method in primary care settings; (2) email makes it possible to reach older patients, but equity issues persist; and (3) commitment varies greatly depending on the clinic, which indicates that the organizational and relational context matters.

We found that a large-scale email campaign using a marketing platform effectively reached a significant number of primary care patients. The high delivery rate of 96.6% (14,758/15,277) is consistent with previous studies [9,18]. Only a few email addresses were incorrect, indicating that patients generally provided valid addresses to their clinic. As expected, reminders were effective for primary care patients recruited by their clinic [10]. Unlike paper questionnaires sent by traditional mail, which remain physically present and serve as a constant reminder, digital questionnaires do not provide a lasting prompt [19]. A high proportion of primary care patients (10,828/14,758, 73.4%) opened the invitation, which is high compared to 35% to 51% rates reported in other studies [20,21]. The average open rate in email marketing campaigns related to health care services sent by the industry is 23% [22]. Within the email marketing industry, open rates above 50% are considered outstanding, typically reflecting the presence of a highly loyal or well-targeted niche audience [23]. This may be attributed to the sender’s email address being from a reputable authority (ie, a primary care practice), which could lead patients to perceive it as more relevant [24] and reduce the likelihood of them mistaking our invitation for spam or a fraudulent solicitation. Indeed, participants may hesitate to open emails from unknown or suspicious addresses [9,25]. Additionally, the clinic used a catchy email subject to capture patients’ attention, which may have contributed to the high open rate [8]. Targeted email campaigns can also enhance recruitment effectiveness, as all invited patients were directly concerned with the study topic [26]. This confirms the organizational feasibility of large-scale recruitment campaigns while imposing minimal burden on clinics.

Our email invitations achieved high engagement indicators, successfully engaging more than 1000 primary care patients. The conversion rate of our email recruitment strategy was 9.7% (1430/14,758), which is higher than the rates typically reported in similar studies (2.9%-4%) [9,24,25,27-29]. General email marketing campaigns in Canada average a conversion rate of less than 3% [8]. This may also be attributed to the clear division of materials into sections, allowing patients to quickly assess the relevance and interest of the information provided [18]. Including multimedia elements, such as illustrations and video clips, likely contributed to higher performance [18]. A total of 1512 clicks were recorded on the patient partner video link, which suggests that this content seems to be of interest to patients. Moreover, simply knowing a patient partner was involved in the study and made a video may have reassured some patients and added external validity [30]. However, the study design does not allow conclusions regarding its causal impact on participation. These findings suggest that while cocreation may enhance acceptability and trust, its contribution should be understood as contextual and supportive rather than as a measurable driver of engagement. This strategy, along with the contribution of patient partners in research and recruitment, is highly recommended as best practice guidelines for patient-oriented research [16,31].

The age range of patients who responded to the survey was broad. Notably, patients aged 70 years or older also answered the questionnaire, suggesting that email can be an effective method for recruiting older patients. This observation aligns with the increasing internet use among older adults in Canada over recent years, as the proportion of those aged 75 years or older using the internet rose from 62% in 2020 to 72% in 2022 [32]. The online survey received a higher response rate from women, which is consistent with comparable studies [33-37]. Additionally, because patients were invited if they had an encounter with their health care professional within the previous 6 months, and women tend to consult primary care more frequently than men, this may have led to a higher proportion of women being invited to participate in the survey [28,38]. For ethical and privacy reasons, our team did not have access to the identity of admissible invited patients and, consequently, to their sociodemographic characteristics. We also observed a low number of responses among people of non-White ethnic origin. Due to the unavailability of data on the ethnic origins of the invited patients, it is not feasible to calculate or compare specific response rates by ethnicity. However, the literature suggests that ethnicity may be a source of nonresponse bias [39,40]. Heerman et al [28] observed similar results, finding that technology-enabled recruitment approaches are not the most efficient for enrolling non-White patients. Therefore, it is essential to consider strategies to enhance the participation of these underrepresented groups in health surveys, primarily because it is known that ethnic groups other than White have a less positive primary care experience [41].

Engagement metrics varied substantially across participating primary care clinics, including differences in open rates, click-through rates, and conversion rates. These variations suggest that a strictly standardized recruitment protocol may not be optimal in all primary care contexts. Although a common email template and overall recruitment strategy were used, clinics adapted key implementation elements—such as timing of invitations, number of reminders, and communication practices—to better align with their organizational context and patient population. This adaptability appears necessary to maintain acceptability and feasibility, highlighting that digital recruitment strategies in primary care may benefit from a balance between standardization (to ensure efficiency and comparability) and local tailoring (to reflect clinic-specific realities and patient expectations). These findings emphasize the importance of context-sensitive implementation when deploying digital recruitment approaches in real-world primary care settings.

This study should be interpreted as a formative, implementation-oriented analysis rather than a test of behavioral theory or recruitment effectiveness. The use of descriptive engagement metrics offers actionable information for research teams seeking to monitor, refine, and adapt digital recruitment strategies over time. Future studies could build on this work by incorporating comparative designs, experimental testing of message components, or qualitative inquiry to elucidate mechanisms of engagement further.

### Limitations

This study had a few limitations that must be underscored. Ideally, participation invitations would have been sent to all patients across clinics simultaneously, and the time between reminders would have been consistent across clinics, allowing for a deeper exploration of factors influencing response rates across clinics. Ideally, to assess the effect of the various strategies embedded in the email, different versions of the message should have been sent to separate groups of patients (for instance, a plain or less visually appealing email or the same message without the patient partner video). However, within the PaRIS study context, it was not feasible to send potentially less-engaging messages to patients, as the primary goal was to maximize participant recruitment. Due to the PaRIS study’s design, objectives, and ethical constraints, we could access only the sociodemographic characteristics of patients who responded to the questionnaire, not those who received the email invitation. Sample representativeness is a major concern, given that only 9.7% (1430/14,758) of the invited patients partially or fully completed the questionnaire. Additionally, staff from some clinics discussed the study with some patients, which may have contributed to a higher conversion rate. However, this intervention was not measured, and its effect cannot be assessed. Our study also revealed variation in indicators across clinics, demonstrating that patients do not all respond similarly to email invitations. These differences may be attributed to the type of clinic (private, public, or university-affiliated) and the characteristics of the patients enrolled in the study. However, our study lacks sufficient power to determine these characteristics.

### Conclusions

This formative, descriptive secondary analysis documents the functioning of email-based recruitment in real-world primary care research settings. Using routinely collected engagement metrics, we show that emails sent on behalf of trusted primary care clinics are a feasible and low-burden approach for reaching patients and supporting participation in web-based surveys. These findings offer practical, implementation-oriented insights for research teams seeking scalable recruitment approaches. They can inform the design, refinement, and ongoing monitoring of digital recruitment strategies in future primary care studies.

## Supplementary material

10.2196/67088 Multimedia Appendix 1 Copy of the standardized email template used to invite eligible patients to participate in the study.

## Abbreviations

OECD: Organisation for Economic Co-operation and Development
PaRIS: Patient-Reported Indicator Survey
REDCap: Research Electronic Data Capture
