# Supplementary material for: Design and Performance of an Email-Based Patient Recruitment Campaign in Primary Care Research: Formative Secondary Analysis
Source: JMIR Hum Factors. 2026 Mar 6;13:e67088. doi: 10.2196/67088 (PMC12978913; doi:10.2196/67088)
Supplement: Multimedia Appendix 1 [file humanfactors-v13-e67088-s001.pdf]

PARIS INTERNATIONAL STUDY: SELF-REPORTED MEASURES BY PEOPLE LIVING WITH CHRONIC DISEASES

QUEBEC PART

WE NEED YOUR EXPERTISE!

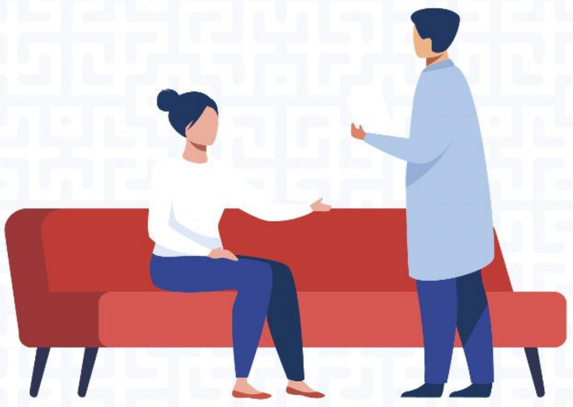

Hello,

Our clinic is one of 135 clinics in Canada participating in the international PaRIS study conducted by the OECD (Organisation for Economic Co-operation and Development). This study focuses on patients' perceptions of their health and care experience. We'd love to hear from you, and your answers will help us improve the quality of services at YOUR clinic.

Here's a short video from Marie-Dominique Poirier, patient partner and co-leader of the study, to tell you all about it! We invite you to view it by clicking on the following link

Partner patient capsule

Interested in taking part in the study?

Please click on the following link to complete the questionnaire:

French questionnaire | English questionnaire

At the start of the questionnaire, you will be asked for your clinic number.
Your clinic number is XX.

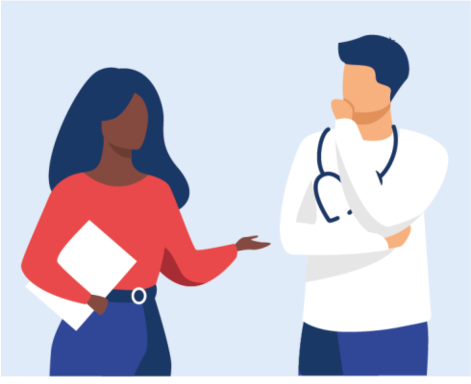

Our aim

Identify common indicators among participants to measure the experience and health of people living withchronic diseases as they perceive them.

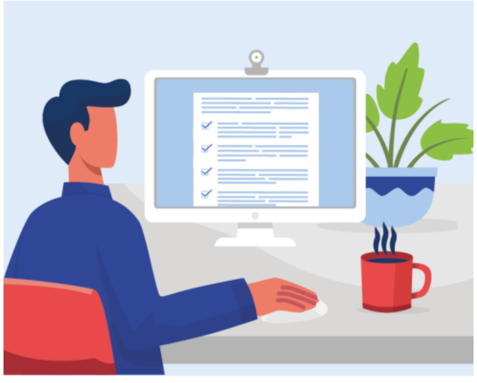

Your contribution

1. Answer a questionnaire online, on paper or by phone
2. Complete an optional 60-90-minute interview

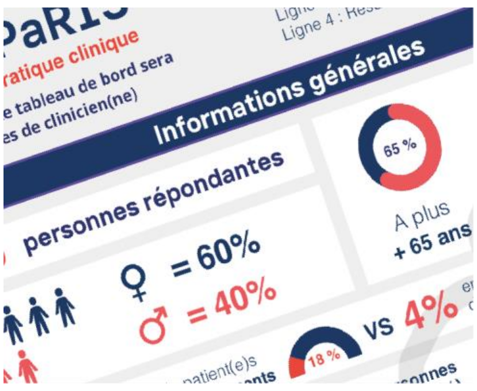

What's in it for you

1. How your clinic compares to otherclinics in Quebec and Canada
2. Contribute to improve the services offered in your clinic
3. Monetary compensation offered for the clinical team's participation in various research-related activities

You can contact Vanessa Vaillancourt, study coordinator, at any time (**vanessa.tremblay-vaillancourt@usherbrooke.ca** or **418-541-5050 ext. 203537**) for further information. She can also send you a **paper copy** of the questionnaire or complete it over the phone.

Finally, if you're interested in learning more about experience and health indicators from the patient's perspective, we're sharing with you some popularization capsules:

What is a PREMs? | What is a PROMs?

Your participation is essential to help us improve our services.
Sincerely yours
[NAME OF CLINICIAN OR CLINIC].
